# CETP/LPL/LIPC gene polymorphisms and susceptibility to age-related macular degeneration

**DOI:** 10.1038/srep15711

**Published:** 2015-10-27

**Authors:** Ya-Feng Wang, Yue Han, Rui Zhang, Li Qin, Ming-Xu Wang, Le Ma

**Affiliations:** 1 School of Public Health, Xi’an Jiao tong University Health Science Center, Xi’an, China; 2 The First Affiliated Hospital, Xi’an Jiao tong University College of Medicine, Xi’an, China

## Abstract

Three high-density lipoprotein (HDL)-related loci have been reported to be associated with age-related macular degeneration (AMD), but the results were inconsistent. In this study, the cholesteryl ester transfer protein (CETP) rs3764261 variant was significantly associated with an increased risk of AMD (odds ratio [OR] = 1.13, 95% confidence interval [CI]: 1.05–1.21, *P* < 0.001), and the hepatic lipase (LIPC) rs10468017 variant was associated with a significantly decreased risk of AMD (OR = 0.81, CI: 0.76–0.86, *P* < 0.001). Individuals carrying the lipoprotein lipase (LPL) rs12678919 polymorphism (A → G) had no significant change in the risk of developing AMD (OR = 1.01, CI: 0.92–1.10, *P* = 0.17). After adjusting for the complement factor H (CFH) gene, both CETP and LPL conferred a significantly increased AMD risk (OR_CETP_ = 1.17, CI: 1.08–1.26, *P* < 0.001; OR_LPL_ = 1.11, CI: 1.01–1.22, *P* = 0.02). Subgroup analysis based on ethnicity revealed a significant association between the CETP variant and AMD in both Americans (OR = 1.12, CI: 1.02–1.23, *P* = 0.01) and Europeans (OR = 1.10, CI: 1.01–1.19*, P* = 0.011). This meta-analysis revealed that both CETP rs3764261 and LIPC rs10468017 polymorphisms were significantly associated with AMD risk. After adjustment for the CFH gene, CETP/LPL conferred a significantly increased susceptibility to the disease, indicating potential interactions among genes in the complement system and the lipid metabolism pathway.

Age-related macular degeneration (AMD) is a progressive neurodegenerative disease and the leading cause of irreversible blindness among individuals aged 65 and older, particularly in western countries^1^,^2^. With the aging population, the number of people with AMD is estimated to increase by approximately 50% by the year 2020 and the burden of this disease is set to grow^3^. The pathological hallmark of the disease is amorphous deposits of protein and lipid, termed drusen, in the retinal pigment epithelium (RPE) and Bruch’s membrane. With progression of these early features, AMD is manifested by geographic atrophy (GA) or the development of choroidal neovascularization (CNV)^4^.

The pathogenesis of AMD is still incompletely understood, and a growing body of evidence has suggested that the accumulation of oxidized lipids seems to play a pivotal role in the development of AMD^5^. Long-term elevated HDL would lead to dysfunctional HDL levels, and pro-oxidant and pro-inflammatory particles that are produced could impair cholesterol efflux and promote LDL oxidation^6^,^7^. Consequently, oxidation products such as peroxidation lipids gradually accumulate in the retina and Bruch’s membrane, thereby initiating inflammation and leading to amorphous deposits, termed drusen, and abnormal angiogenesis^8^. As the major SNPs involved in the HDL pathway, CETP rs3764261, LPL rs12678919 and LIPC rs10468017 have been shown to be associated with AMD in a genome-wide association study (GWAS)^9^. However, previous studies on the reported HDL cholesterol metabolism genes yielded contradictory results, especially in several GWAS; moreover, their genetic susceptibility to AMD varied in diverse populations^10^,^11^,^12^. In addition, as the major AMD-susceptibility gene, perhaps accounting for approximately 30%–50% of AMD patients, CFH might interact with lipid metabolism to affect the disease risk^13^. Such interactions among genes in the complement system and HDL metabolism pathway might be proposed as one potential explanation for the lack of heritability^14^. However, whether the interaction was presence or not, and whether or not these interactions occurred with different genes in the HDL metabolism pathway remained unclear.

Therefore, we conducted a meta-analysis to pool the results of all available studies of CETP rs3764261, LPL rs12678919 and LIPC rs10468017 polymorphisms and the risk of AMD.

## Result

### Study characteristics

**Figure 1:**
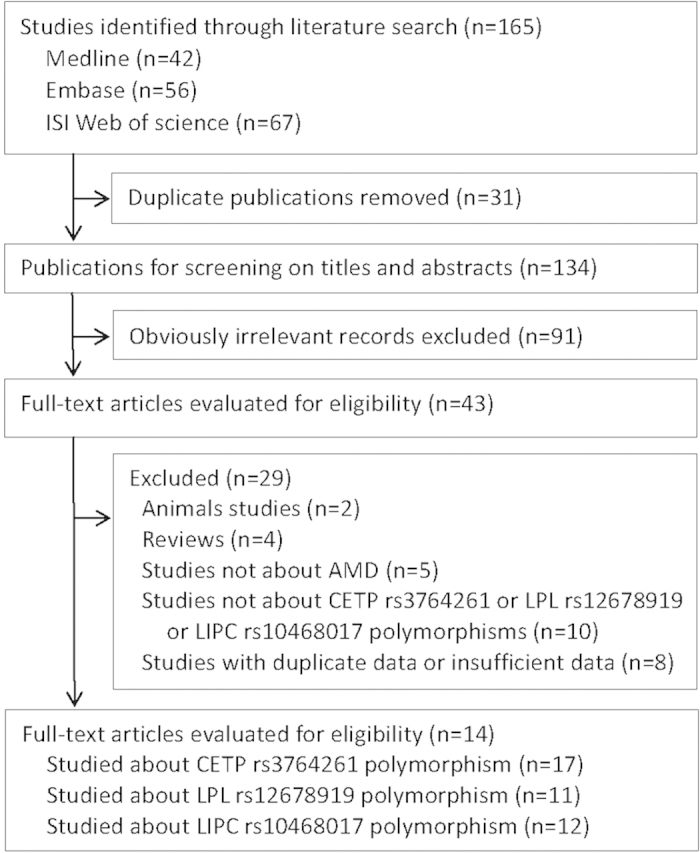
Flowchart for the selection of eligible studies. AMD, age-related macular degeneration.

A total of 165 articles were initially identified from the databases based on our search criteria. After deleting the duplications, 134 articles were left for screening titles and abstracts, with 43 determined to be potentially eligible (Fig. 1). After reviewing the full articles and contacting the authors to obtain the necessary data, 14 articles were retained in our meta-analysis^9^,^10^,^11^,^15^,^16^,^17^,^18^,^19^,^20^,^21^,^22^,^23^,^24^,^25^.

The main characteristics of all included studies are listed in Table 1. Seventeen studies assessed the association between the CETP rs3764261 polymorphism and AMD. Among these, nine studies were conducted on Americans, four on Asians, and four on Europeans. The average age ranged from 65.4 to 80.3 years in case groups and 44.0 to 77.0 years in control groups. In all cases and control subjects, minor A-allele frequencies ranged from 0.32 to 0.37 for Americans, from 0.17 to 0.20 for Asians, and from 0.29 to 0.33 for Europeans. All included studies had case-control designs, and eleven of these studies were GWAS. Thirteen studies adjusted for the top principal component of ancestry (CFH gene), whereas the others did not adjust for it. The genotype frequency was in HWE in the controls for all included studies (*P* > 0.05 for all), except for four studies that did not report the available data. Among the 11 included studies of the LPL rs12678919 polymorphism, seven studies were carried out in Caucasians, and four were performed in Asians. Minor G-allele frequencies ranged from 0.09 to 0.17 in the various populations studied. The average age of subjects ranged from 64.0 years to 81.2 years for cases and from 64.0 years to 77.4 years for controls. There were four GWAS and seven case-control studies. Among the included studies, nine studies adjusted for the CFH gene. In our analysis, no study deviated from HWE in the control groups except for two studies that did not report it. Twelve studies assessed the association between the LIPC rs10468017 polymorphism and AMD. Nine studies were carried out in Caucasians, and three in Asians. Minor T-allele frequencies ranged from 0.11 to 0.35 in different population. Four of the included studies were GWAS. Three studies adjusted for the CFH gene, whereas the others did not adjust for it. The genotype frequency distributions of nine studies were in agreement with HWE, except for the other studies that did not report it.

### Allele comparison

The frequency of the risk allele A in the CETP rs3764261 polymorphism among controls was 30.47% (CI: 26.43–34.52), and it was significantly higher in Americans and Europeans than that in Asians (Americans: 40.11% [CI: 35.11–45.12]; Europeans: 31.74% [CI: 26.94–36.54]; Asians: 18.11% [CI: 12.47–23.74]; all *P* < 0.05). However, there was not a significant difference between the frequency among Americans and Europeans (*P* = 0.26). The T allelic frequency in the LPL rs12678919 variant among controls was 10.17% (CI: 8.86–12.66). No significant difference existed in diverse populations (all *P* > 0.05). The minor allelic frequency in the LIPC rs10468017 variant among controls was 26.55% (CI: 21.43–31.66), and it was significantly higher in Americans and Europeans than that in Asians (Americans: 32.00% [CI: 28.83–35.17]; Europeans: 28.33% [CI: 24.54–32.13]; Asians: 15.57% [CI: 5.32–26.01]; all *P* < 0.05). Similarly, no significant difference was detected between Americans and Europeans (*P* = 0.07).

### CETP rs3764261 polymorphism and AMD risk

**Figure 2:**
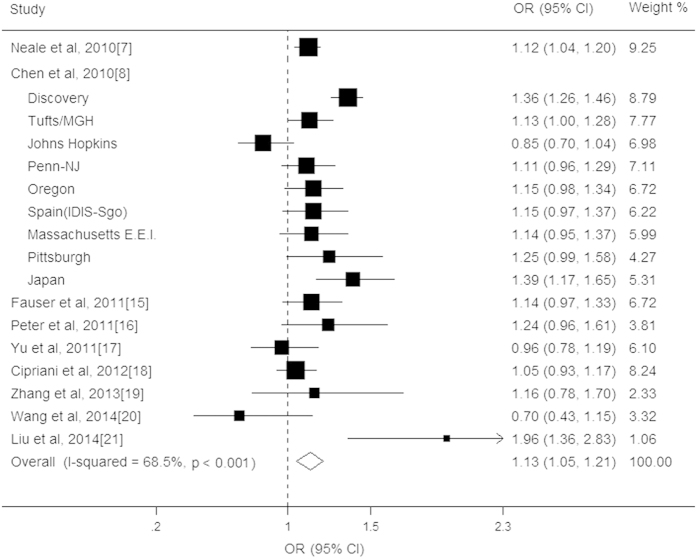
Forest plot on the associations between the CETP rs3764261 polymorphism and age-related macular degeneration under the allelic (C vs. A) genetic model. The boxes and lines indicate the odds ratios (ORs) and their confidence intervals (CIs) on a log scale for each study. The pooled odds ratio is represented by a diamond. The size of the black squares indicates the relative weight of each estimate.

The association between the CETP rs3764261 polymorphism and AMD was investigated in seventeen studies comprising a total of 16888 cases and 15295 controls^9^,^10^,^11^,^17^,^18^,^19^,^20^,^21^,^22^. When all studies were pooled into the meta-analysis, the individuals with the A allele significantly exhibited a 1.13-fold increased risk of AMD compared to individuals with the C allele (OR = 1.13, CI: 1.05–1.21, *P* < 0.001; Fig. 2), with significant heterogeneity (*I*^*2*^ = 68.5%, *P* for heterogeneity 0.012; Table 2). For late AMD, a significant association was observed between the rs3764261 variant and this disease (OR = 1.15, CI: 1.06–1.23, *P* < 0.001), and this locus was also associated with a significant increased risk of CNV (OR = 1.38, CI: 1.04–1.73, *P* < 0.001). After adjustment for the CFH gene, the association of the CETP rs3764261 variant with the increased AMD risk remained significant (OR = 1.17, CI: 1.08–1.26, *P* < 0.001). The result of the stratified analysis by ethnicity revealed that the CETP variant had a significant association with the development of the disease in both Americans (OR = 1.12, CI: 1.02–1.23, *P* = 0.01) and Europeans (OR = 1.10, CI: 1.01–1.19, *P* = 0.011) which disappeared in Asians (OR = 1.24, CI: 0.81- 1.68, *P* = 0.16). However, the pooled OR did not show a significant association among the studies without adjusting for the CFH gene (OR = 1.03, CI: 0.93–1.12, *P* = 0.36). Moreover, no significant association was found when studies had a case-control design excluding GWAS (OR = 1.10, CI: 0.90–1.30, *P* = 0.17). Moreover, statistically significant associations were noted in elderly individuals. Moreover, we carried out a sensitivity analysis by removing each study one at a time and the pooled ORs remained stable indicating that the results were not influenced by any single study. This analysis also revealed that two studies by Chen *et al*. were the main source of heterogeneity. After removing these two studies, the *I*^*2*^ statistics for the ORs decreased from 68.5% to 31.2% (*P* = 0.12), yielding a summary OR of 1.12 (CI: 1.08–1.16, *P* < 0.001) with the same direction of effect. We did not find any evidence for the presence of publication bias in the eligible studies (Begg’s test: *P* = 0.54; Egger test: *P* = 0.45).

### LPL rs12678919 polymorphism and AMD risk

**Figure 3:**
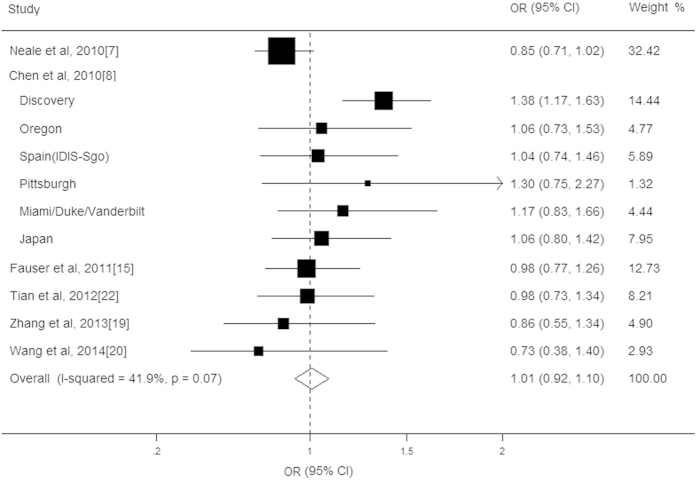
Forest plot on the associations between the LPL rs12678919 polymorphism and age-related macular degeneration under the allelic (A vs. G) genetic model. The boxes and lines indicate the odds ratios (ORs) and their confidence intervals (CIs) on a log scale for each study. The pooled odds ratio is represented by a diamond. The size of the black squares indicates the relative weight of each estimate.

Eleven studies with a total of 13221 cases and 9611 controls assessed the association between LPL rs12678919 polymorphism and AMD risk^9^,^10^,^15^,^17^,^20^,^21^. Unlike the CETP rs3764261 variant, the presence of the LPL rs12678919 polymorphism was not associated with an altered AMD risk (OR = 1.01, CI: 0.92–1.10, *P* = 0.17; Fig. 3), without significant heterogeneity (*I*^*2*^ = 41.9%, *P* = 0.07). In the subtype analysis for AMD, no significant association was found between the LPL rs12678919 variant and a decreased risk of late-stage AMD (OR = 1.04, CI: 0.89–1.18, *P* = 0.13). After adjusting for the CFH gene, the LPL locus showed a significant association with increased AMD risk (OR = 1.11, CI: 1.01–1.22, *P* = 0.02). Subsequently, we also performed stratified analysis by ethnicity, age of case and study design, and no significant associations were found in the other subgroup analysis (Table 2). Most of the observed heterogeneity was contributed to one study with a large sample size. In sensitivity analyses, the exclusion of this study did not appreciably influence the pooled OR. No publication bias was found for Begg’s rank correlation test (*P* = 1.00) or Egger’s linear regression test (*P* = 0.49).

### LIPC rs10468017 polymorphism and AMD risk

**Figure 4:**
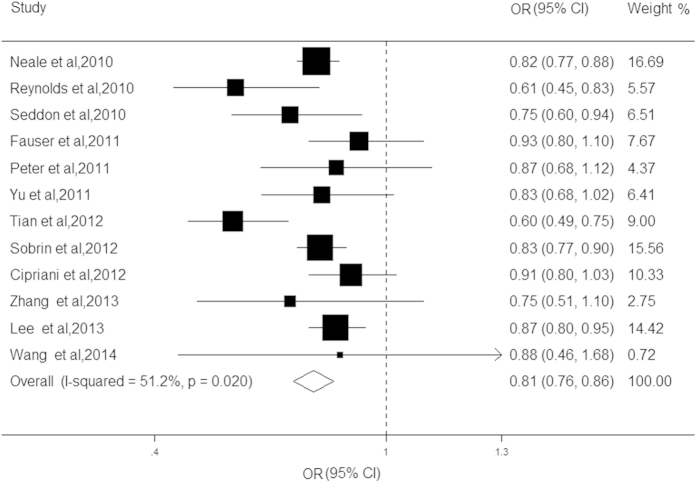
Forest plot on the associations between the LIPC rs10468017 polymorphism and age-related macular degeneration under the allelic (C vs. T) genetic model. The boxes and lines indicate the odds ratios (ORs) and their confidence intervals (CIs) on a log scale for each study. The pooled odds ratio is represented by a diamond. The size of the black squares indicates the relative weight of each estimate.

We subsequently evaluated the relationship between the LIPC rs10468017 polymorphism and the risk of the AMD in twelve studies^9^,^11^,^15^,^16^,^17^,^18^,^19^,^20^,^21^,^23^,^24^,^25^, with a total of 23529 cases and 32757 controls. The present results revealed that the minor allele of this loci was significantly associated with a decreased risk of AMD (OR = 0.81, CI: 0.76–0.86, *P* < 0.001; *I*^*2*^ = 61.0%, *P* = 0.02; Fig. 4). For late AMD, the minor allele of LIPC rs10468017 was found to reduce the risk by approximately 18% (OR = 0.82, CI: 0.79–0.85*, P* < 0.001; *I*^*2*^ = 51.2%, *P* = 0.09). When subgroup analysis was conducted for late AMD subtypes, the summary ORs of rs10468017 were 0.80 (CI: 0.73–0.86, *P* < 0.001) and 0.74 (CI: 0.65–0.82, *P* < 0.001) for CNV and GA, respectively. The stratified analysis revealed that other characteristics including ethnicity, age of case and study design, shared consistency in the direction of effect (OR ranging from 0.64 to 0.91; all *P* < 0.05). After adjusting for the CFH gene, the significant association of the LIPC rs10468017 variant with decreased AMD risk was not appreciably altered (OR = 0.82; CI: 0.77–0.88, *P* < 0.001). In addition, the pooled ORs remained stable by removing each study one at a time, indicating that the results were not influenced by any single study. There was no evidence for the presence of publication bias (*P* > 0.05).

### Trial sequential analysis

**Figure 5:**
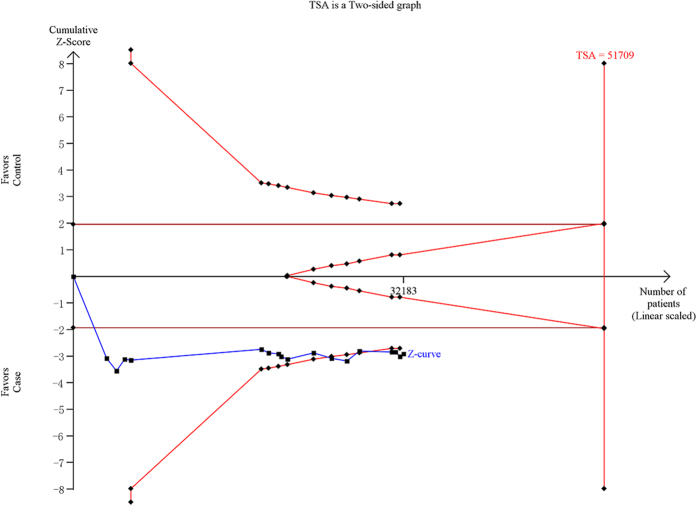
Trial sequential analysis of the CETP rs3764261 polymorphism and age related macular degeneration risk. The diversity-adjusted required information size was based on a relative risk reduction of 13%, an alpha of 5% and a power of 80%. The blue line represents the cumulative Z-score of the meta-analysis. The red straight represent the conventional *P* = 0.05 statistical boundaries. The inward sloping red lines represent the truncated trial sequential monitoring boundaries.

**Figure 6:**
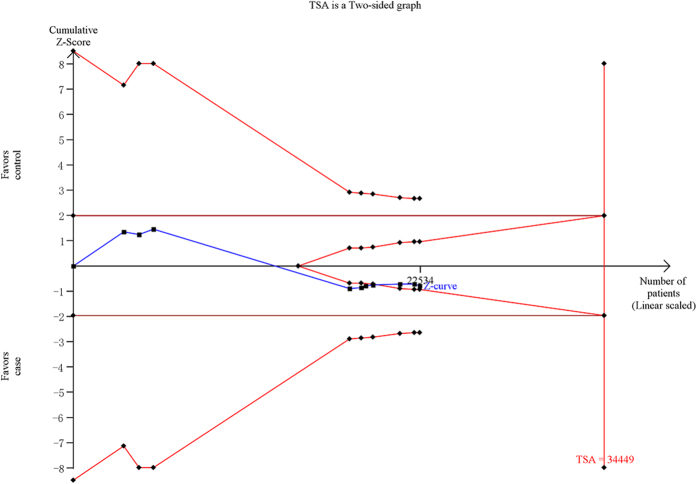
Trial sequential analysis of the LPL rs12678919 polymorphism and age related macular degeneration risk. The diversity-adjusted required information size was based on a relative risk reduction of 10%, an alpha of 5% and a power of 80%. The blue line represents the cumulative Z-score of the meta-analysis. The red straight represent the conventional *P* = 0.05 statistical boundaries. The inward sloping red lines represent the truncated trial sequential monitoring boundaries.

**Figure 7:**
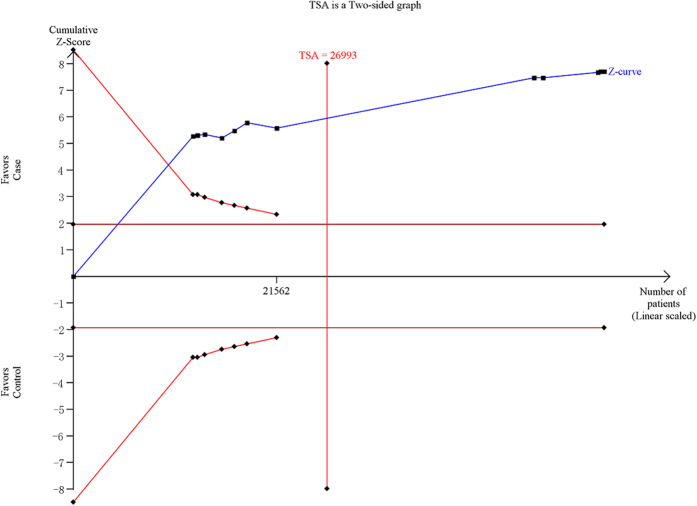
Trial sequential analysis of the LIPC rs10468017 polymorphism and age related macular degeneration risk. The diversity-adjusted required information size was based on a relative risk reduction of 15%, an alpha of 5% and a power of 80%. The blue line represents the cumulative Z-score of the meta-analysis. The red straight represent the conventional *P* = 0.05 statistical boundaries. The inward sloping red lines represent the truncated trial sequential monitoring boundaries.

For CETP rs3764261, the required information size is 51709 subjects using TSA with the settings mentioned in the Methods section. Before the required information size is reached, the cumulative Z-curve (blue line) has crossed the trial sequential monitoring boundaries (inward sloping red lines), indicating that the positive result was confirmed. This resulting trial sequential analysis was shown in Fig. 5. For LPL rs12678919, the result of the trial sequential analysis showed the required information size is 34449 subjects. Before the required information size is reached, the cumulative Z-curve has crossed below the futility boundaries, demonstrating that the power of study is sufficient for this meta-analysis and no further trials are necessary (Fig. 6). For LIPC rs10468017, using the TSA, the required information size is 26993 subjects to demonstrate the issue. The cumulative Z-curve has crossed the trial sequential monitoring boundaries, and the number of participants had exceeded the required information size, suggesting that the significant association of this locus with AMD had been confirmed (Fig. 7). Overall, according to trial sequential analysis program, the results of these three SNPs revealed that the power of study was sufficient for meta-analysis and further relevant trials are unnecessary.

## Discussion

The present meta-analysis showed that the CETP rs3764261 variant was associated with an increased risk of AMD, and the LIPC rs10468017 variant could significantly reduce the susceptibility to AMD. After adjusting for the CFH gene, LPL rs12678919 was also significantly associated with an increased risk of AMD, indicating potential interactions among genes in the complement system and lipid metabolism pathway.

Since 2010, several unknown genes associated with AMD have been identified by large-scale GWAS investigations in the high-density lipoprotein cholesterol (HDL) pathway, whereas all previous genetic variants identified from candidate gene testing were mainly found in the complement factor pathway^9^,^10^. The CETP locus was shown to be associated with an increased risk of AMD by Neale *et al*. and Chen *et al*. but this finding was contradicted by another GWAS^9^,^10^,^11^. In addition, the results of GWAS were inconsistent for the LPL and LIPC genes^9^,^10^.

At a basal state, functional HDL shows anti-inflammatory activity with high levels of antioxidants, active antioxidant proteins, and antioxidant enzymes^26^,^27^. However, the antioxidant and anti-inflammatory activities of HDL become ineffective, when the protective functions of HDL are overwhelmed by inflammation and other factors such as myeloperoxidase-mediated oxidation^28^,^29^,^30^. Therefore, elevated HDL may be converted into dysfunctional, pro-oxidant and pro-inflammatory particles that impair cholesterol efflux and promote LDL oxidation. Consequently, the metabolic components of lipid metabolism could stimulate the angiogenic factors and inflammatory processes by disrupting their regulatory pathways, which are found to be associated with initiation and progression of AMD^28^,^31^,^32^. As the key cholesterol transporter encoded by the CETP gene in the HDL cholesterol pathway, cholesterol ester transfer protein shuttles triglyceride particles from very-low density lipoproteins and low-density lipoproteins to HDL, which results in relatively triglyceride-enriched HDL^33^,^34^. Then, excessive HDL may cause dysfunction and the accumulation of oxidized lipids in the retina, which contribute to the development of AMD. This theory was supported by the present result showing that the CETP rs3764261 variant had a significant association with the increased risk of having AMD. Nevertheless, LPL rs12678919 in the HDL pathway was discovered to be associated with AMD in the same direction as the CETP gene, but this association was not significant. This difference could be because LPL rs12678919 had a critical effect on AMD risk.

After adjusting for the CFH gene, the current study indicates that both the CETP and LPL loci conferred a significantly increased risk of disease, which might contribute to potential interactions among genes in the complement system and lipid metabolism pathway. It has been established that both complement dysregulation and the accumulation of oxidized products of lipid metabolism could modulate genes involving various processes associated with AMD pathology^32^,^35^. As the main genetic polymorphism in the alternative pathway, the CFH risk allele could confer higher complement activation and cell lysis activity, which would bring changes in AMD risk by modulating oxidized lipids accumulation and influencing the expression of angiogenic and proangiogenic molecules^32^,^36^. Under the effect of the CETP and LPL genes in the HDL cholesterol pathway, excessive HDL was produced which could increase the concentration of oxidized phospholipids (oxPLs) in the retina. Due to oxPLs stimulation, increased CFH gene expression could lead to an overactive alternative pathway, which initiates inflammation and activates inflammatory downstream cascades in RPE and macrophages. These outcomes subsequently increase the risk of AMD^35^,^36^. Therefore, CETP and LPL could be the modifier genes of CFH in the development of AMD, indicating that an excess of oxidized lipids links genes with potential interactions to the alternative complement pathway and the HDL metabolic pathway.

In the present meta-analysis, the effect of the CETP rs3764261 variant on AMD might differ in multiethnic populations. In contrast with the absent association in the Asian population, the CETP rs3764261 variant might predispose European and American individuals to develop AMD. It has been observed that the minor allele frequency for this SNP is 0.32 and 0.40 in Europeans and Americans, respectively, which is significantly higher than the frequency of 0.18 observed in Asians. Thus, the consistent effects of these alleles in diverse populations might be due to differences in their genetic backgrounds, suggesting that frequency of the A allele could affect the susceptibility to AMD. However, the different lifestyles in these populations, including smoking, food habits and alcohol consumption, likely also affect the risk of AMD, which might also partly influence our results. Therefore, this finding needs to be considered with thoughtful deliberation, and additional large and carefully designed studies and multivariate analysis that adjusts for confounding factors are necessary.

Previous studies have demonstrated that LIPC encodes hepatic triglyceride lipase, which is expressed in the liver^37^. Under the function of the enzyme hepatic lipase, HDL is partly converted to LDL, and then the pro-oxidant and pro-inflammatory effect of excessive HDL is retarded^24^,^35^,^38^. Ultimately, AMD risk would be decreased in this scenario, which also supports our finding that the locus was protective for AMD.

Several potential limitations should also be acknowledged in interpreting the results from this study. First, as no available cohort studies were found, we could only perform meta-analysis with case-control studies, and this type of study design might be inherently biased by various factors; however, according to the trial sequential analysis, the results of these three SNPs revealed that the power of study was sufficient and the robust evidence might have been confirmed, which adds to the strength of our analysis. Second, both the environmental factors (e.g., smoking, alcohol consumption, and food habits) and other complement factor genes likely affect the risk of AMD, which might also partly influence our results. Our analysis was based primarily on data and information provided from the original literature; however, the included studies did not control for these confounding factors or report sufficient data to analyze the association between CETP/LPL /LIPC genes and AMD adjusted for different environmental factors and other complement factor genes. Thus, the assessment of potential gene-gene or gene-environment interactions was limited, and the possibility that environmental factors and other genes might affect the AMD risk interactively with lipid metabolism could not be excluded in the present study. Further large research studies that allow for the adjustment by these covariates, including genes and environmental factors, should be conducted. Finally, potential publication bias was also a concern. Although we did not observe any apparent evidence of publication bias according to our statistical tests, this analysis does not exclude its possibility. In addition, it was still difficult to fully exclude this problem because there was not a sufficient amount of studies to detect it adequately.

In conclusion, the meta-analysis provides evidence for an association between CETP/LPL/LIPC gene polymorphisms and AMD. Carriage of the CETP rs3764261 A allele would increase the risk of developing AMD, and the LIPC rs10468017 variant was associated with a reduced risk of AMD. No significant association between the LPL rs12678919 polymorphism with AMD was observed in diverse populations. Moreover, this study also identified the conceivable interactions between the CETP and LPL loci and the CFH locus in AMD, indicating that CFH may influence the disease risk interactively with lipid metabolism. Future carefully designed studies with large study populations are warranted to substantiate this association and determine these potential gene-gene and gene-environment interactions in the pathogenesis of AMD.

## Methods

### Search strategy

A systematic literature search of MEDLINE, EMBASE, and ISI web of science databases through December 2014 was conducted to identify all relevant articles involving the association of the CETP and LPL gene with AMD, using the search terms: (“CETP” or “cholesteryl ester transfer protein” or “LPL” or “lipoprotein lipase” or “LIPC” or “hepatic lipase”) and (“AMD or age-related maculopathy or neovascular AMD or exudative AMD or choroidal neovascularization or geographic atrophy or macular degeneration”). The search was not restricted to any language, and we checked the reference lists of retrieved articles and relevant reviews for additional published and unpublished data. When datasets were incomplete for the required data, corresponding authors were contacted for additional information.

### Study selection and data abstraction

We performed an initial screen of identified abstracts and titles to examine all retrieved articles. If the studies did not address the association between CETP/LPL /LIPC genes and AMD, they were excluded. Then we further checked the full texts of the remaining articles for their suitability for the present meta-analysis. The full-text articles of all references selected after the application of these criteria were reviewed by using the same criteria. Although cohort, case-control and cross-sectional studies were considered, no cohort or cross-sectional studies were found. For inclusion, studies included in the meta-analysis had to meet the following screening criteria: (1) the primary outcome was clearly defined as AMD; (2) there were at least two comparison groups, e.g., AMD versus control (non-AMD) groups; and (3) odds ratios (ORs) with their 95% confidence intervals (CIs) or sufficient data to calculate these were reported. When multiple publications reported on the same or overlapping data, only the publication with the most updated data was included.

From each retrieved study, the following data were extracted: the first author name, the year of publication, study design, country, ethnicity, sample size, the mean age of subjects, diagnosis method, classification criteria, type of macular degeneration, the genotype distributions in cases and controls, the methods for genotyping and *P* values for Hardy-Weinberg equilibrium (HWE) in controls. If a study provided several risk estimates, the most completely adjusted estimate was extracted. For studies that provided subcategories of AMD disease status, gradings were collapsed into a single AMD group; meanwhile, ORs of early or late AMD (GA or CNV) were also extracted, respectively. The retrieved studies and extracted data from each included study were independently assessed by two investigators (Y.-F.W. and Y.H.). Any inconsistencies were resolved through consensus with a third author (L. M.) for adjudication.

### Quality assessment

Study quality was independently assessed by two reviewers (Y.-F.W. and Y. H.) with the Newcastle-Ottawa quality assessment scale (NOS) which uses a ‘star system’ to evaluate data quality^39^. The system criteria included three broad perspectives: the selection (four criteria), comparability (one criterion) and exposure (three criteria); the quality scores of studies range from zero (lowest) to nine (highest). A score of five or greater was considered high quality, whereas scores less than four were considered low quality^40^.

### Statistical analysis

HWE was assessed in the control group of each study via the chi-square test. The strength of the association between the CETP, LPL, LIPC polymorphisms and AMD risk was estimated as OR with CI under an allelic genetic model. In case of significant heterogeneity, random effects models were employed to allow for it, otherwise a fixed-effects model was created. The presence of heterogeneity across individual studies was evaluated by the Q statistic and the *I*^*2*^ statistic (*I*^*2*^ > 50% indicated evidence of heterogeneity). Stratified analyses were conducted to explore potential sources of heterogeneity. In addition, we performed sensitivity analysis, after sequentially removing one study at a time, to assess the stability of the pooled results. Potential publication bias was assessed by the application of Egger’s linear regression test, Begg’s rank correlation test and contour-enhanced funnel plots^41^,^42^. STATA version 11.0 (Stata Corp LP, College Station, TX) was used for all statistical analyses. The analysis was conducted independently and in a double blind manner by two investigators (Y.-F.W. and Y.H.). A *P* value <0.05 was considered statistically significant, except for the tests of heterogeneity, Egger’s linear regression and Begg’s rank correlation in which a level of 0.10 was used.

### Trial sequential analysis (TSA)

According to Cochrane Handbook for systematic reviews of interventions, if all available trials are included, meta-analyses are considered to be the best available evidence. However, a meta-analysis may result in type II errors if data are sparse or may increase type I errors and if there is repeated testing for significance when new trials are added^43^,^44^,^45^. Based on these problems mentioned above, the TSA was applied to minimize the random errors and increase the robustness of conclusions^45^,^46^. In our study, the required information size was calculated and TSA monitoring boundaries were built based on an overall type-I error of 5%, a power of 80% and a relative risk reduction assumption of 13%, 10% and 15% for CETP rs3764261, LPL rs12678919 and LIPC rs10468017^43^,^47^. If the cumulative Z-curve has crossed the trial sequential monitoring boundaries or below the futility boundaries before the required information size is reached, robust evidence might have been confirmed and no further studies are necessary, whereas it is necessary to continue doing trials^47^.

## Additional Information

**How to cite this article**: Wang, Y.-F. *et al*. CETP/LPL/LIPC gene polymorphisms and susceptibility to age-related macular degeneration. *Sci. Rep*. **5**, 15711; doi: 10.1038/srep15711 (2015).

## Figures and Tables

**Table 1:** Characteristics of studies included in this meta-analysis.

Surname	Country	Study design	Sample size	Mean Age, y	Diagnosis method	Classification criteria	Type of Case	Adjustment*	MAF	HWE	Study quality**
			(Cases/Contros)	(Case/Control)							
CETP rs3764261 polymorphism											
Neale *et al*. 2010	US	GWAS	6768/5943	79.5/74.2	Fundus photography	CARMS	Late AMD	Yes	NA	NP	High
Chen *et al*. 2010											
Discovery	US	GWAS	2157/1150	78.6/74.1	Fundus photography	CARMS	Late AMD	Yes	0.31	Yes	High
Tufts/MGH	US	GWAS	821/1709	80.3/74.1	Fundus photography	CARMS	Late AMD	Yes	0.36	Yes	High
Johns Hopkins	US	GWAS	641/122	75.5/74.7	Fundus photography	CARMS	Late AMD	Yes	0.40	Yes	High
Penn-NJ	US	GWAS	556/347	79.8/75.6	Fundus photography	CARMS	Late AMD	Yes	0.31	Yes	High
Oregon	US	GWAS	509/253	79.8/74.0	Fundus photography	CARMS	Late AMD	Yes	0.33	Yes	High
Spain(IDIS-Sgo)	Spain	GWAS	348/276	76.7/75.1	Fundus photography	CARMS	Late AMD	Yes	0.27	Yes	High
ME	US	GWAS	386/190	76.0/75.4	Fundus photography	CARMS	Late AMD	Yes	0.32	Yes	High
Pittsburgh	US	GWAS	170/143	69.9/76.7	Fundus photography	CARMS	Late AMD	Yes	0.32	Yes	High
Japan	Japan	GWAS	654/333	74.8/74.2	Fundus photography	CARMS	Late AMD	Yes	0.17	Yes	High
Fauser *et al*. 2011	Dutch and German	Case-control	1201/562	75.9/72.7	Fundus photography	CIRCL	AMD	Yes	0.34	NP	High
Peter *et al*. 2011	European	Case-control	146/1269	74.5/73.6	Fundus photography	WARMGS	Late AMD	No	0.32	Yes	High
Yu *et al*. 2011	US	Case-control	1082/221	79.3/77	Fundus photography	CARMS	Early and late AMD (GA/CNV)	No	0.41	Yes	High
Cipriani *et al*. 2012	UK	GWAS	893/2199	78.6/44.5	Fundus photography	ICGS	Late AMD	No	0.33	NP	High
Zhang *et al*. 2013	China	Case-control	157/204	65.4/69.0	Fundus photography	NR	CNV	Yes	0.17	NP	High
Wang *et al*. 2014	China	Case-control	199/99	65.8/66.2	Fundus photography	NR	Late AMD	No	0.21	Yes	High
Liu *et al*. 2014	China	Case-control	200/275	75.3/74.3	Fundus photography	NR	CNV	Yes	NA	Yes	High
LPL rs12678919 polymorphism											
Neale *et al*. 2010	US	GWAS	6768/5943	79.5/74.2	Fundus photography	CARMS	Late AMD	Yes	NA	Yes	High
Chen *et al*. 2010											
Discovery	US	GWAS	2157/1154	78.6/74.1	Fundus photography	CARMS	Late AMD	Yes	0.10	Yes	High
Oregon	US	GWAS	507/252	79.8/74.0	Fundus photography	CARMS	Late AMD	Yes	0.09	Yes	High
Spain(IDIS-Sgo)	Spain	GWAS	245/217	76.7/75.1	Fundus photography	CARMS	Late AMD	Yes	0.17	Yes	High
Pittsburgh	US	GWAS	174/149	69.9/76.7	Fundus photography	CARMS	Late AMD	Yes	0.08	Yes	High
MDV	Spain and US	GWAS	699/246	75.7/68.4	Fundus photography	CARMS	Late AMD	Yes	0.09	Yes	High
Japan	Japan	GWAS	647/323	74.8/74.2	Fundus photography	CARMS	Late AMD	Yes	0.12	Yes	High
Fauser *et al*. 2011	Dutch and German	Case-control	1201/562	75.9/72.7	Fundus photography	CIRCL	AMD	Yes	0.10	NP	High
Tian *et al*. 2012	China	Case-control	467/462	67.9/65.0	Fundus photography	AREDS	Late AMD	No	0.09	Yes	High
Zhang *et al*. 2013	China	Case-control	157/204	65.4/69.0	Fundus photography	NR	CNV	YES	0.14	NP	High
Wang *et al*. 2014	China	Case-control	199/99	65.8/66.2	Fundus photography	NR	Late AMD	No	0.11	Yes	High
LIPC rs10468017 polymorphism											
Neale *et al*. 2010	US	GWAS	6768/5943	79.5/74.2	Fundus photography	CARMS	Late AMD	Yes	0.30	Yes	High
Reynolds *et al*. 2010	US	Case-control	318/140	81/76	Fundus photography	CARMS	Late AMD(GA/CNV)	No	0.35	NP	High
Seddon *et al*. 2010	US	Case-control	545/275	73.5/67.5	Fundus photography	CARMS	Late AMD(GA/CNV)	No	0.32	NP	High
Fauser *et al*. 2011	Dutch and German	Case-control	1201/562	75.9/72.7	Fundus photography	CIRCL	AMD	Yes	0.30	Yes	High
Peter *et al*. 2011	European	Case-control	146/1269	74.5/73.6	Fundus photography	WARMGS	Early and late AMD	No	0.28	Yes	High
Yu *et al*. 2011	US	Case-control	1082/221	79.3/77.0	Fundus photography	CARMS	Early and late AMD (GA/CNV)	No	0.34	Yes	High
Tian *et al*. 2012	China	Case-control	467/462	67.9/65.0	Fundus photography	AREDS	Late AMD(GA/CNV)	No	0.17	Yes	High
Sobrin *et al*. 2012	US	GWAS	7977/19374	69.0/68.0	Fundus photography	CARMS	Late AMD(GA/CNV)	No	NR	NP	High
Cipriani *et al*. 2012	UK	GWAS	893/2199	78.6/44.5	Fundus photography	ICGS	Late AMD	No	0.27	Yes	High
Zhang *et al*. 2013	China	Case-control	157/204	65.4/69.0	Fundus photography	NR	CNV	Yes	0.19	Yes	High
Lee *et al*. 2013	US	GWAS	199/99	79.3/72.6	Fundus photography	ICGS	Late AMD	No	0.29	Yes	High
Wang *et al*. 2014	China	Case-control	199/99	65.8/66.2	Fundus photography	NR	Late AMD	No	0.11	Yes	High

AREDS, Age-related eye disease study; CARMS, Clinical age-related maculopathy staging system; CETP, Cholesteryl ester transfer protein; CFH, Complement factor H; CIRCL, Cologne image reading center and laboratory; CNV, Choroidal neovascularization; GA, Geographic atrophy; GWAS, Genome wide association study; HWE, Hardy-Weinberg equilibrium; ICGS, International classification and grading system; LIPC, Hepatic lipase; LPL, Lipoprotein lipase; MAF, Minor allele frequency; MDV, Miami/Duke/Vanderbilt; ME, Massachusetts E.E.I.; NA , Not available; NR, Not reported; WARMGS, Wisconsin age-related maculopathy grading system.

^*^Adjust for CFH gene.

^**^Study quality was judged based on Newcastle-Ottawa Scale.

**Table 2:** Meta-analysis of the association between the CETP rs3764261/LPL rs12678919/ LIPC rs10468017 polymorphisms and age-related macular degeneration risk

Variables	N	Cases/Controls	Pooled OR (CI)	*P*	
				Heterogeneity	Meta-regression
CETP rs3764261 variant					
Late AMD	16	15687/14733	1.15(1.06,1.23)	<0.001	NA
CNV	3	1498/651	1.38(1.04,1.73)	0.19	
GA	1	641/122	1.13(0.82,1.44)	NA	
Ethnicity					
Americans	9	13090/10078	1.12(1.02,1.23)	<0.001	0.27
Europeans	4	2588/4306	1.10(1.01,1.19)	0.59	
Asians	4	1210/911	1.24(0.81,1.68)	0.003	
Age of case					
≥75	12	15562/13247	1.14(1.10,1.18)	<0.001	0.47
<75	5	1326/2048	1.21(1.07,1.35)	0.043	
Study design					
GWAS	11	13903/12665	1.15(1.06,1.24)	<0.001	0.98
Case-control	6	2985/2630	1.10(0.90,1.30)	0.029	
Adjusting for CFH gene					
Yes	13	16888/15295	1.17(1.08,1.26)	<0.001	0.17
No	4	2477/3992	1.03(0.93,1.12)	0.23	
LPL rs12678919 variant					
Late AMD	10	12020/9049	1.04(0.89,1.18)	0.046	NA
CNV	1	157/204	0.86(0.47,1.26)	NA	
Ethnicity					
Americans	4	9606/7498	1.12(0.78,1.45)	0.002	0.65
Europeans	2	1446/779	1.00(0.80,1.20)	0.79	
Europeans/Americans	1	699/246	1.17(0.75,1.58)	NA	
Asians	4	1470/1088	1.24(0.81,1.68)	0.70	
Age of case					
≥75	5	10878/8128	1.06(0.84,1.27)	0.007	0.73
<75	6	2343/1583	1.00(0.84,1.16)	0.71	
Study design					
GWAS	7	11197/8284	1.10(0.91,1.29)	0.019	0.26
Case-control	4	2024/1327	0.94(0.77,1.10)	0.81	
Adjusting for CFH gene					
Yes	9	12398/8846	1.11 (1.01,1.22)	0.017	0.06
No	2	823/765	0.81(0.50,1.12)	0.70	
LIPC rs10468017 variant					
Late AMD	11	22328/32195	0.82(0.79,0.85)	0.090	NA
CNV	6	10546/20676	0.80(0.73,0.86)	0.36	
GA	5	10389/20472	0.74(0.65,0.82)	0.13	
Ethnicity					
Americans	6	20466/27962	0.82(0.79,0.86)	0.21	0.84
Europeans	3	2240/4030	0.91(0.83,0.99)	0.91	
Asians	3	823/765	0.64(0.52,0.75)	0.49	
Age of case					
≥75	6	14038/11074	0.84(0.80,0.88)	0.089	0.17
<75	6	9491/21683	0.79(0.73,0.84)	0.071	
Study design					
GWAS	4	19414/29525	0.84(0.81,0.88)	0.45	0.26
Case-control	8	4115/3232	0.76(0.69,0.82)	0.041	
Adjusting for CFH gene					
Yes	3	6967/6042	0.82(0.77,0.88)	0.85	0.32
No	9	16562/26715	0.81(0.74,0.87)	0.007	

CETP, Cholesteryl ester transfer protein; CFH, Complement factor H; CI, Confidence interval; CNV, Choroidal neovascularization; GA, Geographic atrophy; GWAS, Genome-wide association study; LIPC, Hepatic lipase; LPL, Lipoprotein lipase; N, Number of studies; NA , Not available; OR, Odds ratio.
